# The maternal-to-zygotic transition is a critical window for PFOA-induced disruption of developmental programming

**DOI:** 10.3389/fcell.2026.1887853

**Published:** 2026-08-26

**Authors:** Zainab Afzal, Vandana Veershetty, Evan E. Pittman, Charles Hatcher, Deepak Kumar

**Affiliations:** 1 The Julius L. Chambers Biomedical and Biotechnology Research Institute, North Carolina Central University, Durham, NC, United States; 2 Department of Biological and Biomedical Sciences, North Carolina Central University, Durham, NC, United States; 3 Department of Pharmaceutical Sciences, North Carolina Central University, Durham, NC, United States

**Keywords:** maternal-zygotic transition, NAMs (new approach methodologies), PFAS, PFOA, perfluorooctanoic acid, zebrafish, development

## Abstract

Early embryogenesis is governed by precisely timed gene regulatory programs that coordinate cell fate specification, tissue patterning, and morphogenesis. The maternal-to-zygotic transition (MZT) represents a pivotal developmental milestone during which regulatory control shifts from maternally deposited transcripts to activation of the zygotic genome. Disruption of this transition has the potential to alter developmental trajectories with lasting consequences. Per- and polyfluoroalkyl substances (PFAS), environmentally persistent contaminants, have been linked to developmental abnormalities, yet their impact on core embryonic gene regulatory networks especially with exposure during MZT is not well understood. Using zebrafish (*Danio rerio*), a tractable vertebrate model and New Approach Methodology (NAM), we investigated how PFAS exposure during the MZT alters early developmental programming. Embryos were exposed starting at different times before and within the MZT time window and collected at 24 h post-fertilization (hpf) for transcriptomic analysis. Targeted qRT-PCR revealed dysregulation of genes controlling transcriptional activation, lineage specification, proliferation, and differentiation. Whole-transcriptome RNA sequencing (RNA-seq) further identified widespread perturbations in gene networks governing transcriptional regulation, cell signaling, and embryonic morphogenesis. Temporal analysis revealed that exposure beginning at 3.5 hpf, followed by 8 hpf, corresponding to early zygotic genome activation and near completion of zygotic activation, respectively, resulted in the greatest differential gene expression changes at 24 hpf. Consistent with these early gene regulatory perturbations, larvae exposed starting at 8 hpf also exhibited altered behavior at 5 days post-fertilization. Together, these findings demonstrate that PFAS exposure during MZT disrupts the establishment of embryonic gene regulatory networks, linking environmental toxicant exposure to altered developmental patterning and organismal outcomes. This work underscores the vulnerability of early developmental transitions to environmental perturbation and positions MZT as a critical window of susceptibility during development.

## Introduction

Poly- and perfluoroalkyl substances (PFAS) are a large class of synthetic chemicals widely used in industrial processes and consumer products for more than 60 years due to their surfactant properties and resistance to heat, oil, and water (Buck et al., 2011; kissa, 2001). Although production and use of several long-chain PFAS have declined over the past 2 decades, their extreme chemical stability has resulted in widespread environmental persistence and ongoing exposure in humans, livestock, and wildlife, with potential consequences for ecosystem and intergenerational health (Bauer et al., 2026; Fenton et al., 2021; Liu et al., 2026; Mellouk et al., 2025; Meng et al., 2022; Satbhai et al., 2025; Agents, 2024). The strength of the carbon–fluorine bond renders PFAS highly resistant to environmental degradation (Smart BE, 2024), leading to their detection in air, soil, drinking water, and biological samples including human serum and cord blood (Cheng et al., 2025; Falls et al., 2026; Hu et al., 2016; Monroy et al., 2008; Zhai, 2015; Zhi, 2024). Epidemiological and toxicological studies have linked PFAS exposure to hepatotoxicity, endocrine disruption, neurodevelopmental deficits, altered embryonic development, oxidative stress, and carcinogenic outcomes (Gaillard et al., 2025; Kebieche et al., 2025; Luebker et al., 2005; Olsen et al., 2007; Qi et al., 2023; Rawtani et al., 2026; Satbhai et al., 2025). Among PFAS compounds, Perfluorooctanoic acid (PFOA) has been of particular concern due to its historical prevalence in industrial applications and persistence in the environment (Nakayama, 2019; Paul et al., 2009).

To investigate how PFOA exposure affects early vertebrate development and to determine whether specific developmental windows exhibit heightened sensitivity, we utilized Zebrafish (*Danio rerio*) embryos. Zebrafish embryos are a powerful vertebrate model and a widely used new approach methodology (NAM) (Ball et al., 2025; Kalueff, 2014), for studying the developmental and neurobehavioral impacts of environmental toxicants because of their rapid external development, genetic conservation with humans, optical transparency, and well-characterized behavioral paradigms (Hill et al., 2005; Howe, 2013; Orger and de Polavieja, 2017). As PFAS can bioaccumulate and cross the placental barrier (Gützkow, 2012), exposure during embryonic development particularly concerning. Zebrafish embryos provide a powerful system to study these effects because they develop externally, allowing precise control of exposure concentrations and timing. In addition, large numbers of synchronous embryos can be studied as they undergo a well-defined maternal-to-zygotic transition (MZT) approximately 3–8 h post-fertilization, during which maternal transcripts are degraded and the zygotic genome becomes transcriptionally active (Giraldez et al., 2006; Schier, 2007; Vastenhouw et al., 2019). This developmental transition provides a tractable system for examining how environmental exposures influence gene regulation during early embryogenesis and for identifying critical windows of exposure vulnerability.

Previous studies have identified transcriptional responses associated with PFAS exposure (Blake et al., 2022; Bline et al., 2025; Britton et al., 2024; Cao et al., 2023; Jantzen et al., 2016; Rericha et al., 2024). To further characterize molecular responses especially during early development, we combined targeted analysis of candidate genes with unbiased transcriptomic profiling to capture both pathway-specific and genome-wide transcriptional changes following PFOA exposure. Using physiologically relevant concentrations of PFOA (Boone, 2019; Stein et al., 2013), we started exposure to zebrafish embryos at multiple timepoints during early developmental windows encompassing the MZT and for all exposed embryos evaluated transcriptional changes at 24 h post-fertilization, a stage when major organ systems are forming (Kawasaki et al., 2017). Bulk RNA sequencing was used to identify global gene expression changes, disrupted pathways, and regulatory networks associated with developmental toxicity, enabling us to determine which developmental window is most sensitive to PFOA exposure.

To link molecular changes with organismal outcomes, we also integrated transcriptomic analysis with developmental and behavioral assessments. Larval locomotor behavior was quantified at 5 days post-fertilization by measuring distance traveled, swimming velocity, and spatial preference within the recording well. In larval zebrafish, spatial preference within an arena can reflect anxiety-like responses following environmental stimuli. This systems-level approach combining transcriptomic, developmental, and behavioral analyses helps us to move beyond phenotype-based toxicity assessments to identify molecular mechanisms by which PFAS disrupt early vertebrate development. Ultimately, these findings contribute to a deeper understanding of the biological consequences of early-life PFAS exposure and may inform environmental risk assessment and public health strategies.

## Materials and methods

### Animal husbandry of adult zebrafish

Adult zebrafish (*D. rerio*, wild-type AB strain) were maintained on a 14:10 h light: dark cycle in embryo media (reverse osmosis water buffered with sea salt, Instant Ocean©) at 27 °C–30 °C in a recirculating system (Aquaneering, San Diego, CA, United States). Water pH was maintained between 7.0 and 7.5. Fish were fed an irradiated diet (Zeigler Bros Inc., Catalog # AH271) twice daily and supplemented with live brine shrimp (E.G., Artemia, INVE AQUACULTURE Inc.). All zebrafish procedures were approved by the Institutional Animal Care and Use Committee (IACUC) at North Carolina Central University (Protocol number D16-00378; approved 06-01-2022).

### Spawning and embryo collection

Adult zebrafish were bred in a 2:4 male-to-female ratio in spawning tanks separated overnight. At 09:00 the next morning, dividers were removed. Immediately following spawning within the 15 min window, fertilized embryos were collected and rapidly sorted under a stereomicroscope. Embryos designated for the one-cell treatment group were transferred directly into treatment medium immediately after collection, allowing chemical exposure to begin during the one-cell developmental stage before the first cleavage occurred. Remaining embryos were sorted into different dishes, and we treated them at different time points along MZT. Embryos were then maintained in treatment medium under standard laboratory conditions (incubated at 28 °C).

### Chemical exposure

Perfluorooctanoic acid (PFOA) (Sigma-Aldrich, Catalog #171468-5G, ≥95% purity) was dissolved in zebrafish embryo medium to prepare a 1000 μg/L stock solution. Working solutions were prepared fresh for each experiment. Embryos from the same clutch were either kept as untreated controls or exposed to 700 ng/L PFOA, a concentration selected to reflect physiologically relevant environmental exposure levels. Control untreated embryos were maintained in embryo medium containing 0.1% methylene blue. For exposure experiments, embryos from the same clutch were sorted into separate Petri dishes and treated with 700 ng/L PFOA in embryo medium 0.1% methylene blue, according to their assigned exposure time. Exposures were initiated at four developmental stages before and within the maternal-to-zygotic transition (MZT): the 1-cell stage (pre-MZT), 3.5 h post-fertilization (hpf), 5 hpf, and 8 hpf. Embryos from all treatment groups were collected at 24 hpf for RNA extraction and transcriptomic analysis. For behavioral assays, larvae were maintained in treatment conditions until 5 days post-fertilization (dpf). Chemical exposures were carried out in new Petri dishes (Fisherbrand 100 mm Petri Dishes with Clear Lid, Catalog # FB0875713), and treatment solutions were changed daily until behavioral testing at 5 dpf.

### RNA extraction for q-RT-PCR and bulk sequencing

At 24 h post-fertilization (hpf), embryos from each treatment and control group were manually dechorionated, and the same pooling and RNA extraction procedures were followed for both PCR and RNA sequencing. For each sample type, four biological replicates were generated, with each replicate consisting of 20 pooled embryos (n = 4 biological replicates per condition). Each replicate containing 20 embryos was homogenized in 100 µL of TRIzol reagent (Invitrogen, Catalog #15596018). Total RNA was extracted using the Direct-zol™ RNA Miniprep Kit (Zymo Research, Catalog #R2073-A), including an on-column DNase treatment to remove genomic DNA contamination. RNA concentration and purity were assessed using a NanoDrop One spectrophotometer (Thermo Fisher Scientific).

For targeted gene expression analysis, complementary DNA (cDNA) was synthesized from purified RNA. Quantitative real-time PCR (qPCR) was performed to assess the expression of selected genes associated with biological processes including developmental toxicity, stress response, DNA damage, and neuromuscular development. Gene expression levels were normalized to the reference gene *gapdh*, and relative expression changes between PFOA-treated and control groups were calculated using the 2^−ΔΔCt analysis method. Statistical analysis of qRT-PCR data was performed in R. For each target gene, expression levels in the untreated control group were compared independently with each treatment group using two-tailed Student’s *t*-tests. A * was denoted if p < 0.05, ** if p < 0.01, *** if p < 0.001, and “ns” if not statistically significant.

In parallel, for RNA sequencing four biological replicates, purified RNA samples were shipped on dry ice to Novogene for transcriptomic analysis. RNA-seq libraries were prepared using the Illumina TruSeq Stranded mRNA Library Prep protocol and sequenced on the Illumina NovaSeq platform to generate 150-bp paired-end reads. Initial bioinformatics processing included quality control, alignment to the *D. rerio* reference genome (GRCz11), and gene-level quantification. Differential gene expression analysis was performed in Novamagic using EdgeR algorithm, with significance thresholds set at an adjusted p-value (false discovery rate, FDR) ≤ 0.05 and an absolute log_2_ fold-change ≥2.

### Statistical tests and behavior analysis

Statistical analyses for tail twitch and survival (alive/dead) ratios were performed in R using the ggpubr package. A one-way ANOVA was used to assess overall differences among groups, followed by pairwise Student’s *t*-tests comparing each PFOA exposure group to the untreated control group. P-values for multiple comparisons were adjusted using the Holm method. Statistical significance was defined as p ≤ 0.05.

Neurobehavioral outcomes were assessed at 5 days post-fertilization (dpf) using the DanioVision Observation Chamber (Noldus Information Technology). For assesment, 24 larvae per group, free of visible morphological abnormalities, were placed individually into wells of a 24-well plate. To minimize potential plate and positional effects, the placement of treatment groups within the 24-well plates, as well as the order in which plates were recorded, was randomized between experiments. All behavioral recordings were conducted in the same DanioVision chamber under standardized environmental conditions, including controlled temperature and automated light stimuli. Locomotor activity was recorded using EthoVision XT software across four sequential conditions: no stimulus (60 s), light stimulus (60 s), no stimulus (60 s), and mechanical vibration stimulus (60 s). Total distance moved and zone preference (center vs. periphery) were quantified as measures of locomotor activity and anxiety-like behavior, respectively. All behavioral data was tested using Shapiro-wilk test for normality. When it was determined data does not follow a normal distribution, the Kruskal-Wallis test was done to determine if there are significant differences between the five untreated and treatment groups, followed by Dunn *post hoc* test to identify specifically which pairwise groups differ significantly. All data plots were visualized in R using the ggplot2 package. A * was denoted if p < 0.05, ** if p < 0.01, *** if p < 0.001, and “ns” if not statistically significant.

## Results

### Embryo viability and early phenotypic responses to PFOA exposure

To establish a physiologically relevant exposure dose, we did a literature search through both environmental and human biomonitoring data. A survey of treated water from 25 drinking water treatment plants across the United States reported a median combined PFAS concentration of 19.5 ng/L, with a maximum of 1,100 ng/L (Boone, 2019). PFOA concentrations measured in serum samples from children have reported mean values between 92,500 and 97,300 ng/L (Stein et al., 2013). Based on these benchmarks and a review of the existing literature, an environmentally relevant and within reported contaminated water ranges, 700 ng/L was selected as the exposure dose for all subsequent experiments.

**FIGURE 1:**
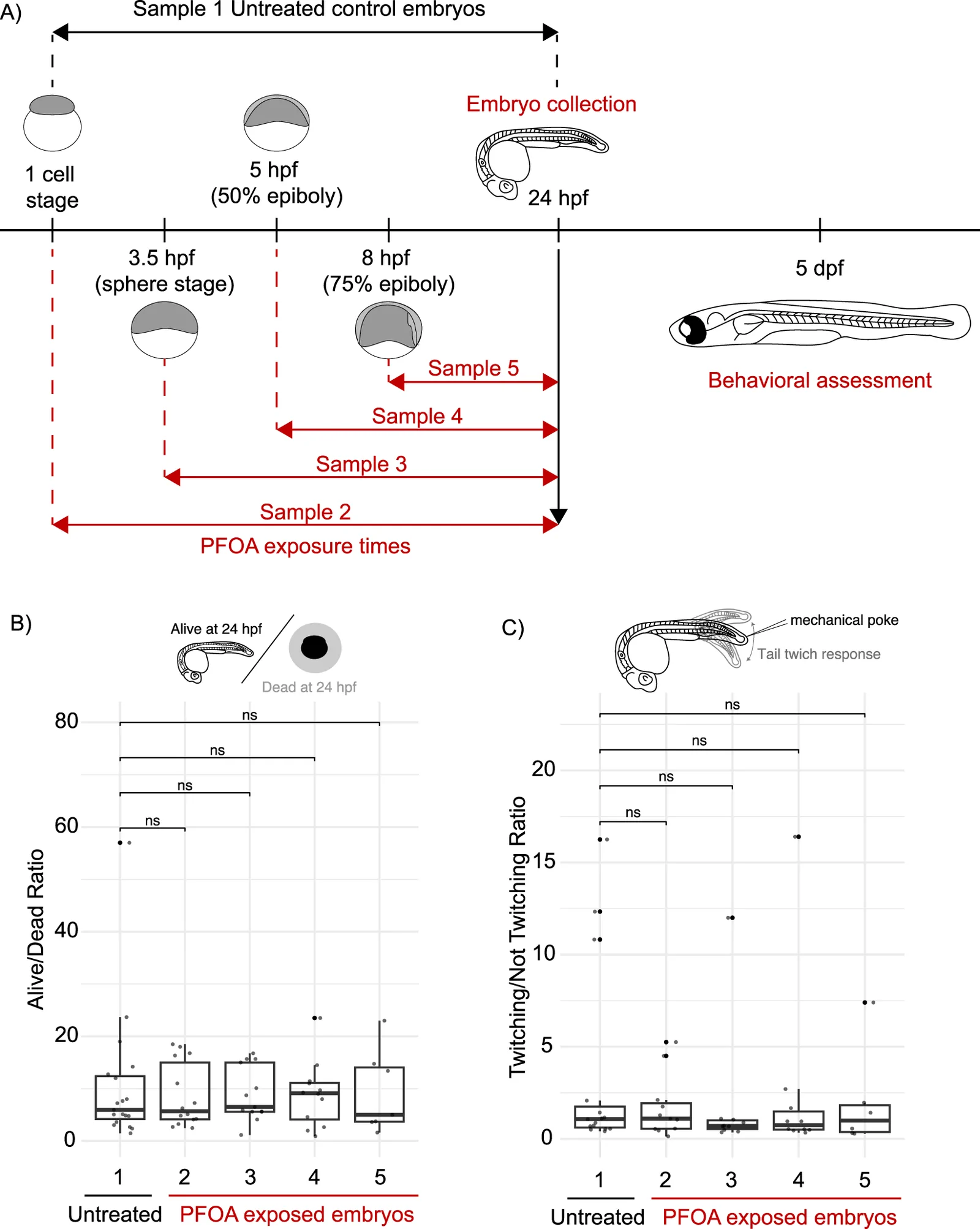
**(A)** Schematic illustrating PFOA exposure time points during the maternal-to-zygotic transition (MZT) and the embryo fixation point at 24 hpf for all subsequent experiments. Embryos were treated at the indicated developmental stages and remained exposed until collection at 24 hpf. **(B)** Box plots to depict embryo viability following PFOA treatment compared to untreated control embryos. **(C)** Box plots to depict manual assessment of tail twitching in response to mechanical stimulation in treated embryos compared to untreated controls (control n = 945; 1-cell treatment n = 578; 3.5 hpf treatment n = 621; 5 hpf treatment n = 228; 8 hpf treatment n = 300). Statistical significance was calculated using using Student’s t-tests with Holm-adjusted p-values for multiple testing correction; p-value <0.05 was considered significant and comparisons were denoted as “ns” where differences were not significant.

To identify the most vulnerable developmental window, embryos were exposed to PFOA at multiple time points spanning the maternal-to-zygotic transition (MZT), 1-cell (pre-MZT), 3.5 hpf (early blastula), 5 hpf (mid-blastula transition), and 8 hpf (gastrulation onset). In all experimental conditions, PFOA exposure was initiated at different stages within the MZT window, and embryos were allowed to develop and subsequently fixed at 24 hpf (Figure 1A). Viability was assessed at 24 hpf by quantifying the ratio of live to dead embryos, where dead embryos included those exhibiting arrested development or morphological abnormalities. No significant difference in the live-to-dead ratio was observed between PFOA-treated and untreated control groups across any treatment time point (Figure 1B).

At 24 hpf, zebrafish embryos normally exhibit a tail twitch response to mechanical stimulation, reflecting proper neuromuscular junction formation. To assess proper neuromuscular junction formation, each embryo was gently prodded with forceps and scored as responsive or non-responsive by noting whether it twitched or did not twitch. Data were collected across multiple independent biological replicates (spawning events). The total number of embryos analyzed for each experimental group represents the pooled sample across all biological replicates, whereas each point shown in the boxplots corresponds to a single biological replicate (one spawning event). Because embryo yield varied between spawning events and not every replicate produced the same number of embryos, the total number of embryos analyzed differs slightly among experimental groups. Although PFOA-treated embryos showed a trend toward an increased proportion of non-responsive embryos relative to untreated controls, suggesting a potential delay in neuromuscular junction development or formation, this difference did not reach statistical significance (Figure 1C).

### PFOA exposure disrupts signaling pathways and transcriptional programs essential for embryonic development

To investigate the molecular consequences of PFOA exposure during early embryogenesis, we first performed targeted qRT-PCR analysis at 24 hpf using a panel of genes selected based on their established roles in developmental processes known to be highly active during early embryogenesis. These candidate genes were chosen to represent key regulatory pathways controlling neural induction, embryonic patterning, cell proliferation, DNA damage responses, and muscle differentiation, all of which are essential for normal organogenesis and have previously been implicated in PFAS-mediated developmental toxicity.

**FIGURE 2:**
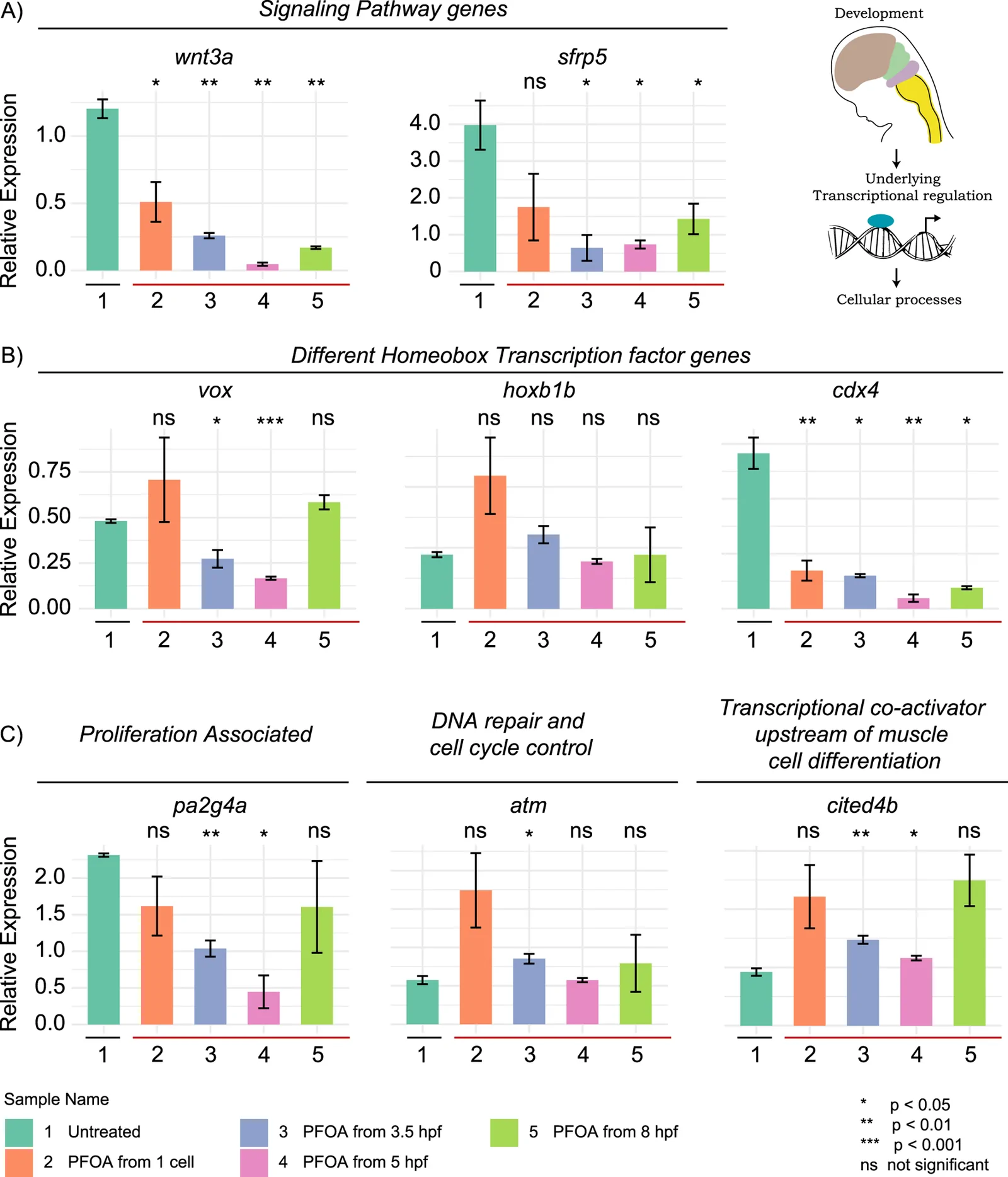
Gene expression changes following PFOA exposure during early development. **(A)** Expression of signaling pathway genes assessed by qRT-PCR at 24 hpf following low-concentration PFOA exposure. Relative gene expression is shown as bar graphs with standard deviation. For each exposure group, N = 20 pooled embryos across five treatment conditions, including a control. **(B)** Expression of selected homeobox transcription factor genes assessed at 24 hpf following PFOA exposure. **(C)** Expression of genes associated with cell proliferation, DNA damage response, and muscle differentiation following PFOA exposure. All statistical analyses were performed using a paired student t-test. Statistical significance is indicated by asterisks: * equals *p* < 0.05, ** equals p < 0.01, *** equals *p* < 0.001, and “ns” denotes non-significant differences.

As the central nervous system is particularly susceptible to developmental toxicants, we first examined the Wnt signaling pathway, which plays a critical role in brain development (Patapoutian and Reichardt, 2000). We observed that PFOA exposure significantly disrupted WNT signaling, as evidenced by marked decreases in the expression of both *wnt3a* and the Wnt antagonist *sfrp5* (Figure 2A), suggesting a broader perturbation of Wnt pathway regulation rather than a simple loss of pathway activity.

Given that Wnt signaling operates upstream of numerous transcriptional programs, that some PFAS compounds can directly bind to DNA (Qin et al., 2024), and that PFAS exposure has been epidemiologically linked to neurodevelopmental deficits (Chen et al., 2026; Wu et al., 2024), we next investigated whether PFOA extends its disruptive effects to embryonic patterning. Towards this, we investigated whether PFOA exposure effects transcriptional regulation during early brain development, focusing on homeobox genes which are highly conserved regulators of downstream targets critical for anterior-posterior and dorsal-ventral axis patterning during development (Figure 2B). Expression of *Hoxb1b*, a transcription factor essential for hindbrain segmentation and anterior-posterior patterning of the central nervous system (McClintock et al., 2002), was not significantly altered by PFOA exposure. In contrast, *Vox*, a homeobox gene involved in dorsal-ventral brain patterning (Imai et al., 2001), was significantly downregulated, particularly when exposure began at 3.5 or 5 hpf, indicating a timing-dependent sensitivity to PFOA. *Cdx4*, which functions in posterior body patterning, hindbrain development, and intestinal formation (Skromne et al., 2007), was consistently downregulated across all PFOA treatment conditions, suggesting that PFOA alters the expression of key homeobox transcription factors in a time-dependent manner, potentially contributing to delayed or abnormal neural development.

To determine whether these transcriptional disruptions also translate into broader cellular dysfunction, we examined the expression of genes involved in cell proliferation, DNA damage response, and cell cycle regulation (Figure 2C). Embryos exposed to PFOA at the 1-cell stage showed no significant changes in these markers; however, exposure initiated at 3.5 hpf resulted in significant reductions in the expression of *pa2g4a*, a proliferation marker (Liu et al., 2006), and significant increase in *atm*, a DNA damage response gene (Khanna et al., 2001), by 24 hpf, suggesting that PFOA exposure during the maternal-to-zygotic transition may impair cellular proliferation and DNA repair mechanisms during a critical window of neural tissue development. Given that these proliferative and DNA repair deficits are likely to have downstream consequences on tissue formation and considering the observed trend toward reduced tail twitch responses in PFOA-treated embryos, a behavior dependent on functional neuromuscular junctions, we further investigated whether these effects extended beyond the nervous system to muscle development. Expression analysis of *cited4b*, a gene involved in muscle cell differentiation (Devakanmalai et al., 2013), revealed significant upregulation following PFOA exposure, potentially reflecting compensatory or dysregulated neuronal and muscle differentiation signaling programs.

### PFOA exposure across the maternal-to-zygotic transition induces stage-dependent transcriptional disruption

**FIGURE 3:**
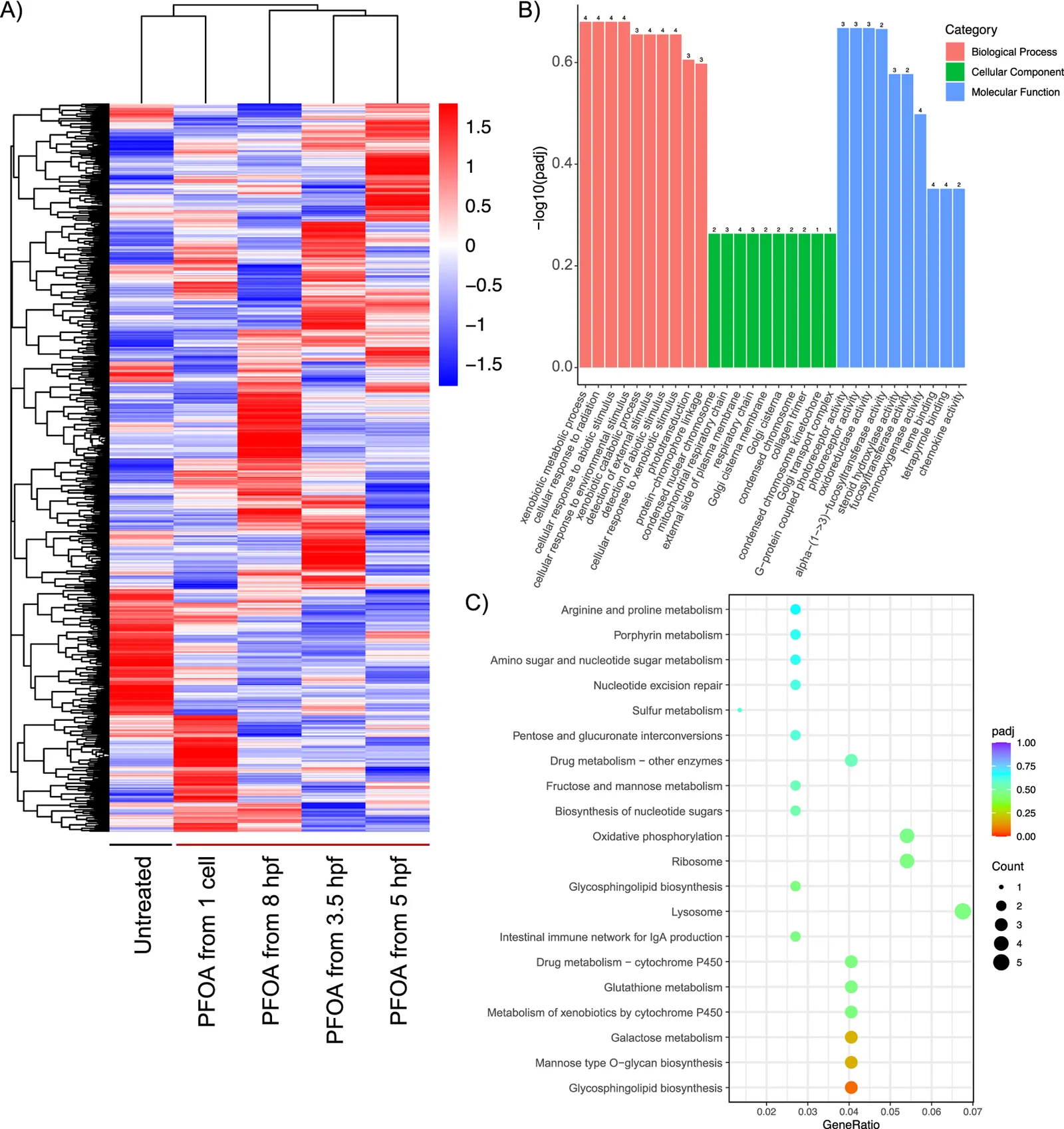
**(A)** Heatmap visualization of global transcriptional changes between untreated and PFOA-treated samples. Gene expression changes were clustered using log2(FPKM+1) values. Red indicates higher expression levels, while blue indicates lower expression levels, with the color gradient reflecting decreasing log2(FPKM+1) values from high to low. **(B)** Bar graph of Gene Ontology (GO) term enrichment analysis of differentially expressed genes (DEGs) identified across all PFOA exposure treatments compared to untreated controls. Enrichment is shown across three categories: Biological Process (BP), Cellular Component (CC), and Molecular Function (MF). **(C)** Dot plot of KEGG pathway enrichment analysis comparing all PFOA exposure treatments to untreated controls, highlighting major pathway disruptions associated with exposure. Dot color represents statistical significance, while dot size indicates the number of DEGs mapped to each KEGG pathway.

While targeted gene expression analysis provided insight into specific developmental pathways affected by PFOA, we sought to obtain an unbiased, genome-wide view of the transcriptional disruptions occurring as a consequence of exposure across the MZT. To achieve this, bulk RNA sequencing was done on all 5 samples, untreated controls and 4 different treatment times before and during MZT. Differential expression analysis was performed to identify genes whose expression differed between untreated controls and PFOA-treated embryos following exposure initiated at these distinct developmental stages. The RNA-seq analysis was designed to characterize how the transcriptional response to PFOA varies across stage-specific windows of exposure during early embryonic development, rather than quantifying the overall activation of the zygotic genome during the maternal-to-zygotic transition (MZT). The heatmap shows gene expression profiles across samples with red rows being genes overexpressed and blue being genes under expressed genes (Figure 3A). These findings demonstrate that the transcriptional response to PFOA differs depending on the developmental stage at which exposure is initiated, highlighting the maternal-to-zygotic transition as a critical window of susceptibility to PFOA-induced molecular perturbation. Comparing across the PFOA exposure conditions, several gene clusters downregulated in controls are overexpressed, and gene clusters exhibiting higher expression are getting downregulated with exposure. When we compare all PFOA exposure conditions together against untreated controls, Gene Ontology (GO) analysis revealed enrichment across all three ontology categories (Figure 3B). Within biological processes (BP), enriched terms included xenobiotic metabolic processing, cellular responses to abiotic and environmental stimuli, phototransduction, and detection of external stimuli, suggesting that PFOA exposure broadly activates stress response and environmental sensing pathways. Cellular component (CC) terms were enriched for Golgi apparatus-associated structures, including Golgi cisterna and Golgi membrane, as well as condensed chromosomes and the mitochondrial respiratory chain, indicating potential disruptions to intracellular organization and energy metabolism. Molecular function (MF) terms highlighted enrichment in receptor activity, including G-protein coupled photoreceptors and photoreceptor activity, alongside transferase activities such as fucosyltransferase, steroid hydroxylase, and alpha-(1→3)-fucosyltransferase, as well as heme and tetrapyrrole binding and chemokine activity, pointing to disruptions in signal transduction, protein glycosylation, and immune-related molecular functions.

Additionally, KEGG pathway analysis revealed enrichment in several functionally related categories (Figure 3C). Metabolic pathways were prominently represented, including amino acid metabolism (arginine and proline metabolism), carbohydrate metabolism (fructose and mannose metabolism, pentose and glucuronate interconversions, amino sugar and nucleotide sugar metabolism), and biosynthesis of nucleotide sugars, suggesting broad disruption of core metabolic processes. Enrichment in oxidative phosphorylation and glutathione metabolism further points to mitochondrial dysfunction and impaired oxidative stress response following PFOA exposure. Notably, multiple xenobiotic and drug metabolism pathways were enriched, including metabolism of xenobiotics by cytochrome P450, drug metabolism via cytochrome P450, and drug metabolism by other enzymes, indicating activation of detoxification machinery in response to PFOA. Glycan biosynthesis pathways were also enriched, including glycosphingolipid biosynthesis and mannose type O-glycan biosynthesis, alongside porphyrin metabolism and nucleotide excision repair, suggesting potential disruptions to lipid signaling, DNA repair, and post-translational protein modification. Finally, enrichment of the intestinal immune network for IgA production pathway suggests that PFOA exposure may also impact immune system development and function as the embryo develops.

Having established the global transcriptional impact of PFOA, we next dissected each developmental exposure window independently to determine how the molecular response across MZT results in distinct changes to identify stage-specific windows of developmental vulnerability. At the 1-cell pre-MZT stage window of exposure, PFOA induced bidirectional transcriptional changes, with upregulated pathways associated with lipid storage, spliceosomal assembly, and endoderm formation, while pathways governing basal transcription initiation and innate immune complement activation were suppressed, suggesting early metabolic reprogramming and immune dysregulation as an immediate consequence of PFOA exposure. Disruptions to cell cycle progression, DNA damage repair, and oxidative phosphorylation were also evident at this earliest stage, indicating that PFOA begins to interfere with fundamental cellular processes from the very onset of development.

As developmental exposure window progressed to 3.5 and 5 hpf, pathway enrichment patterns suggested the simultaneous activation of compensatory mechanisms alongside ongoing developmental disruption. Wnt signaling showed stage-specific upregulation, potentially reflecting a compensatory response to the Wnt downregulation observed at 24 hpf by qRT-PCR, while DNA damage response and cell proliferation pathways were also upregulated. Conversely, muscle contraction and behavior regulation pathways were suppressed. At 5 hpf exposure window specifically, downregulation of miRNA-mediated gene silencing and upregulation of mitotic spindle organization suggest active remodeling of the transcriptional landscape coinciding with the MZT.

With the 8 hpf exposure window, PFOA exposure produced its most pronounced transcriptional effects, characterized by strong immune activation and broad metabolic suppression. Upregulated pathways included chromatin organization and T cell receptor signaling, reflecting escalating stress-induced epigenetic and immune responses, while ATP metabolism, oxidative phosphorylation, and mitochondrial respiratory chain function were significantly downregulated alongside suppression of Wnt signaling, DNA damage checkpoints, and muscle development programs. These 8 hpf exposure window findings are consistent with the stage-dependent exposure window alterations observed in the targeted qRT-PCR analysis, with both approaches indicating disruption of developmental signaling, cellular proliferation, stress-response pathways, and neuromuscular development following PFOA exposure. Together, these complementary datasets demonstrate that PFOA exposure starting during MZT suggests progressive disruption of core transcriptional, metabolic, and developmental programs in a stage-dependent manner.

### Most critical time windows of PFOA exposure during MZT

**FIGURE 4:**
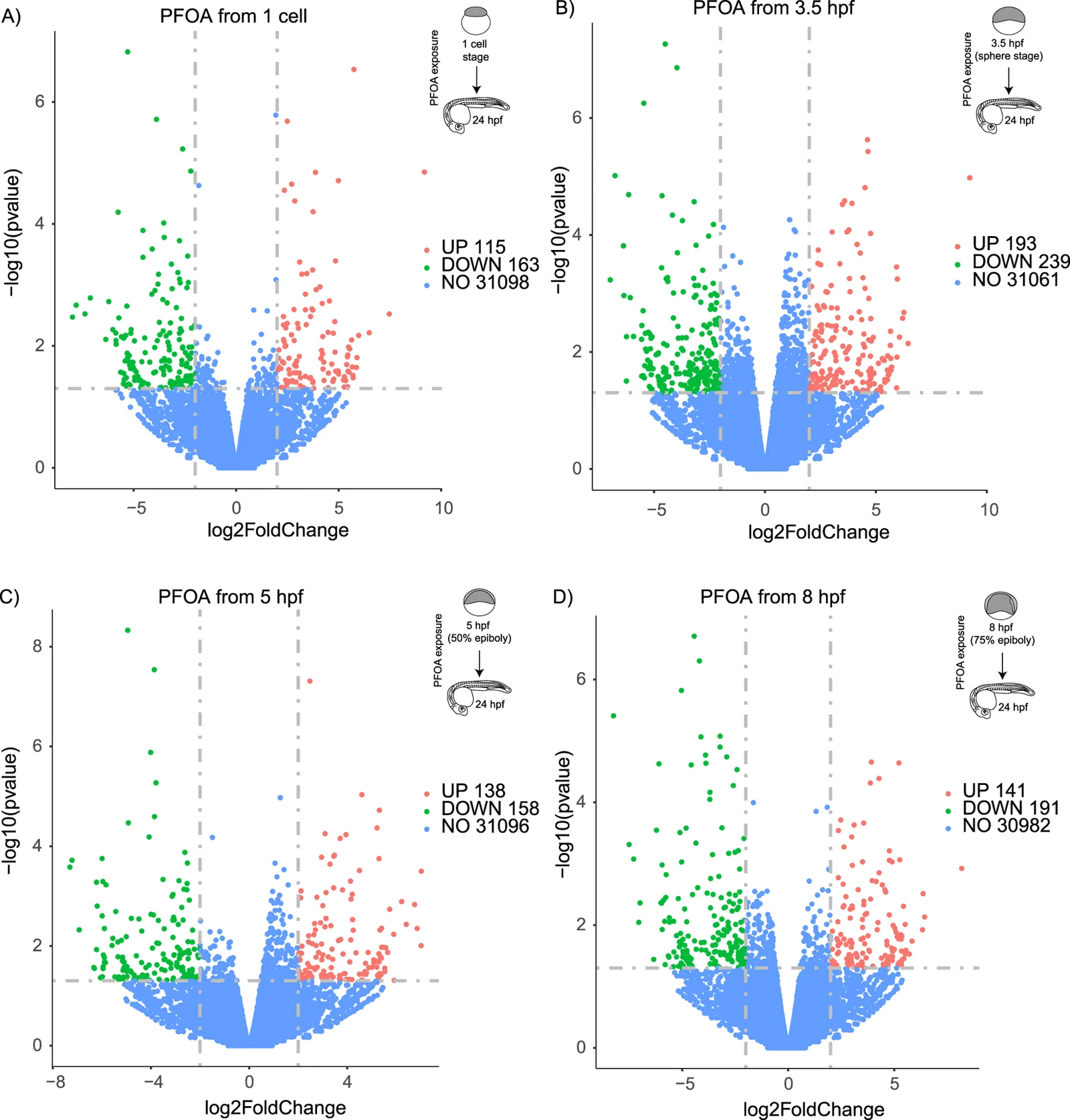
Volcano plots showing differentially expressed genes (DEGs) across all PFOA exposure conditions compared to untreated controls. **(A)** Exposure beginning at the 1-cell stage. **(B)** Exposure beginning at 3.5 hpf. **(C)** Exposure beginning at 5 hpf. **(D)** Exposure beginning at 8 hpf. Non-significant genes are shown in blue, while significantly upregulated genes are shown in red and significantly downregulated genes are shown in green. Significance is defined by a log2 fold change threshold of ≥2 (equivalent to a 4-fold change in expression) in either direction relative to untreated controls.

Comparative analysis of volcano plots across all maternal-to-zygotic transition (MZT) exposure windows revealed that embryos exposed to PFOA beginning at 3.5 hpf exhibited the greatest number of differentially expressed genes relative to untreated controls, with the highest combined total of upregulated and downregulated genes, followed by the 8 hpf exposure group (Figure 4). Although the total number of genes detected at 24 hpf was comparable across all exposure groups, the number of significantly differentially expressed genes revealed that embryos exposed beginning at 3.5 hpf and 8 hpf exhibited the greatest transcriptional perturbations, identifying these stages as the most transcriptionally vulnerable windows of exposure during MZT. To further characterize the biological consequences of exposure at each developmental window, Gene Ontology (GO) enrichment analyses were performed across the categories of biological process, cellular component, and molecular function, alongside KEGG pathway analysis to identify disrupted cellular pathways and developmental processes (Supplementary Figures S1–S4).

**FIGURE 5:**
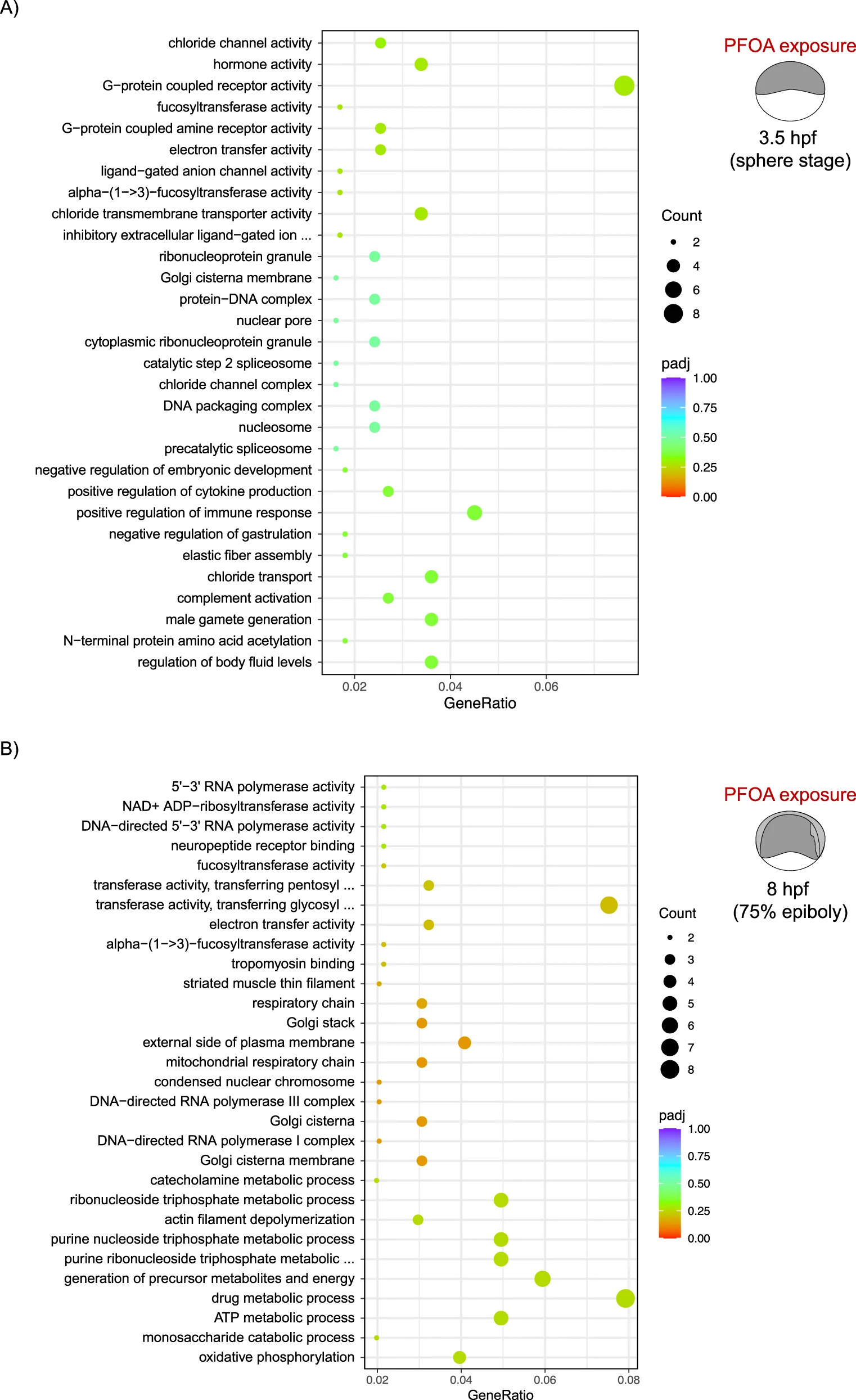
**(A)** Dot plot of Gene Ontology (GO) term enrichment analysis of differentially expressed genes (DEGs) identified in 3.5 hpf exposure treatment compared to untreated control. Enrichment is shown across three categories: Biological Process, Cellular Component, and Molecular Function. **(B)** Dot plot of Gene Ontology (GO) term enrichment analysis of differentially expressed genes (DEGs) identified in 8 hpf exposure treatment compared to untreated control. Enrichment is shown across three categories: Biological Process, Cellular Component, and Molecular Function.

To better understand the molecular basis underlying this heightened sensitivity, GO and KEGG pathway analyses were examined in greater detail for the 3.5 hpf and 8 hpf exposure conditions (Figure 5). Embryos exposed to PFOA beginning at 3.5 hpf showed enrichment of biological processes associated with embryonic development, ion homeostasis, and immune regulation. Cellular component terms indicated disruptions in chromatin organization and RNA processing machinery, including enrichment of nucleosome- and spliceosome-associated components. Molecular function analysis further revealed dysregulation of membrane signaling and ion transport pathways, including chloride channel activity, G protein-coupled receptor signaling, and fucosyltransferase activity (Figure 5A). Consistent with these findings, KEGG pathway analysis identified the spliceosome and p53 signaling pathways as among the most significantly enriched pathways, suggesting that PFOA exposure during this developmental window disrupts RNA processing, genomic stability, and DNA damage response pathways (Supplementary Figure S2B). Additional enrichment of autophagy, oxidative phosphorylation, PPAR signaling, and cardiac muscle contraction pathways further indicates widespread disruption of cellular metabolism and organ-specific developmental programs. Together, these findings suggest that 3.5 hpf represents a particularly sensitive developmental window during which PFOA exposure interferes with core transcriptional, metabolic, and stress-response mechanisms essential for normal embryogenesis.

In contrast, PFOA exposure beginning at 8 hpf predominantly affected pathways associated with mitochondrial metabolism, energy production, transcriptional regulation, and cellular signaling (Figure 5B; Supplementary Figure S4A). Enriched biological process terms included oxidative phosphorylation, ATP metabolic processes, generation of precursor metabolites and energy, and purine ribonucleoside triphosphate metabolism, indicating substantial disruption of mitochondrial bioenergetics and cellular energy homeostasis. Enrichment of mitochondrial respiratory chain and electron transfer activity terms further supported impairment of oxidative metabolism and mitochondrial function. Additional enrichment of drug metabolic processes and catecholamine metabolism suggested altered stress-response and detoxification pathways following exposure. Cellular component analysis identified significant enrichment of Golgi cisterna membranes, Golgi stacks, condensed nuclear chromosomes, and RNA polymerase I and III complexes, suggesting disruptions in intracellular trafficking, chromatin organization, and transcriptional machinery. Molecular function terms, including tropomyosin binding, actin filament depolymerization, neuropeptide receptor binding, and multiple glycosyltransferase activities, further indicated dysregulation of cytoskeletal organization, signaling pathways, and protein modification processes. KEGG pathway analysis supported these observations, highlighting oxidative phosphorylation and RNA polymerase-associated pathways as major targets of PFOA exposure (Supplementary Figure S4B). Table 1 highlights the top differentially expressed genes identified following PFOA exposure beginning at 8 hpf, one of the most transcriptionally vulnerable windows identified in this study. Many of these genes are associated with conserved developmental pathways including vesicle trafficking, chromatin regulation, neural development, calcium signaling, and mitochondrial metabolism. Several of these genes are also linked to maternal or early zygotic transcriptional programs during the maternal-to-zygotic transition. Collectively, these findings demonstrate that exposure beginning at 8 hpf disrupts mitochondrial activity, energy metabolism, transcriptional regulation, and developmental signaling pathways during a critical stage of embryonic development.

**TABLE 1:** Top differentially expressed upregulated and downregulated genes identified by RNA-seq analysis in the 8 hpf PFOA treatment condition, including corresponding human orthologs, conserved developmental functions, and associations with zygotic genome activation during early embryogenesis.

Comparison	Gene	Human ortholog	Role during development	MZT association
8 hpf PFOA vs. untreated, top upregulated genes	*ap1m3*	AP1M3	Protein trafficking between Golgi and endosomes (Chapuy et al., 2008; Shin et al., 2021)	Maternally deposited transcripts (Lee et al., 2013)
	*fam168b*	FAM168B	CNS development, neurite outgrowth, and myelination (Zhang et al., 2020)	Maternal-zygotic transition time ∼5 hpf (Baia Amaral et al., 2024)
	*cbx8b*	CBX8	Chromatin organization and progenitor differentiation (Creppe et al., 2014)	Maternally deposited transcripts (Lee et al., 2013)
	*tnfsf14a*	TNFSF14	Muscle differentiation (Waldemer-Streyer and Chen, 2015) and regulation of neurite growth (Gavalda et al., 2009)	Post-MZT immune activation (Farrell et al., 2018)
8 hpf PFOA vs. untreated top downregulated genes	*cpne3*	CPNE3	Calcium-dependent membrane signaling expressed in neutrophil precursors (Cowland et al., 2003)	Maternally deposited transcripts (Lee et al., 2013)
	*p2rx5*	P2RX5	ATP-gated ion channel with roles in neural, muscle, and cardiac development (Guo et al., 2013; Ruppelt et al., 2001)	Zygotic activation (Lee et al., 2013)
	*mrpl49*	MRPL49	Mitochondrial ribosomal protein associated with neurodevelopment, hearing function, and energy metabolism (Thomas et al., 2025)	Zygotic activation time ∼6 hpf (Baia Amaral et al., 2024)
	*uqcrq*	UQCRQ	Embryonic energy metabolism through mitochondrial oxidative phosphorylation (Barel et al., 2008)	Maternal-zygotic transition time ∼4 hpf (Baia Amaral et al., 2024)

### PFOA-induced disruption development is associated with anxiety-like behavior at the larval stage

The transcriptomic and pathway analyses described above demonstrate that PFOA exposure during the maternal-to-zygotic transition disrupts multiple biological processes critical for normal embryonic development, including mitochondrial metabolism, stress-response signaling, transcriptional regulation, and neurodevelopmental pathways. Given the importance of these processes in nervous system development and organismal homeostasis, we next sought to determine whether these early molecular disruptions resulted in measurable behavioral abnormalities at later developmental stages. To assess the potential long-term functional consequences of embryonic PFOA exposure, larval zebrafish were evaluated for locomotor and anxiety-like behaviors at 5 dpf using the DanioVision tracking system under sequential no-stimulus, light stimulus, and mechanical stimulus conditions.

**FIGURE 6:**
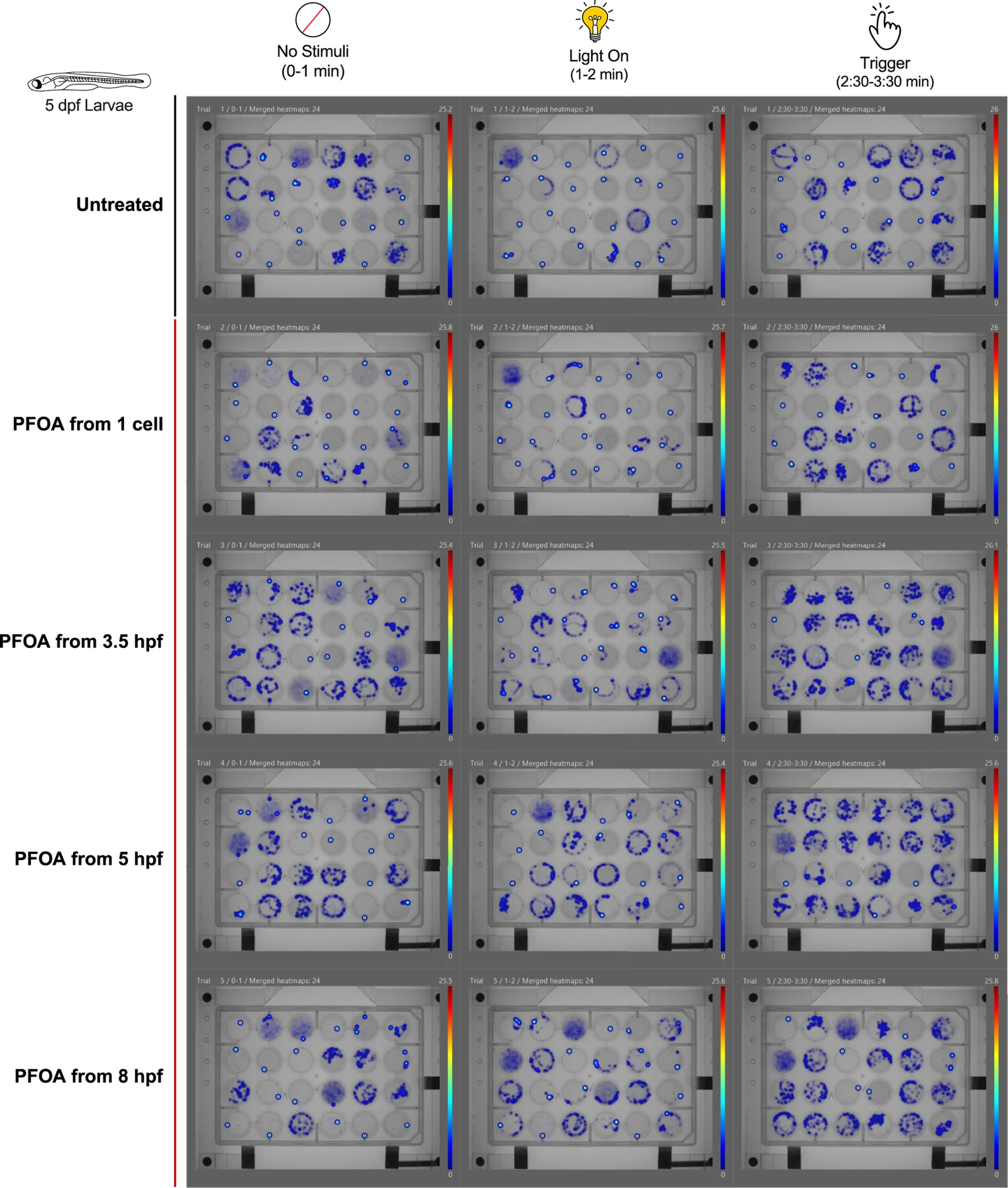
Heatmap visualization of zebrafish larval behavior at 5 dpf under stimulus and no-stimulus conditions. One larva per well was recorded for 3 min in a 24-well plate. The heatmap represents the movement patterns and distance traveled by each larva within the well. Blue traces indicate larval movement, while bright puncta with a red center represent locations where the larvae remained stationary for extended periods.

**FIGURE 7:**
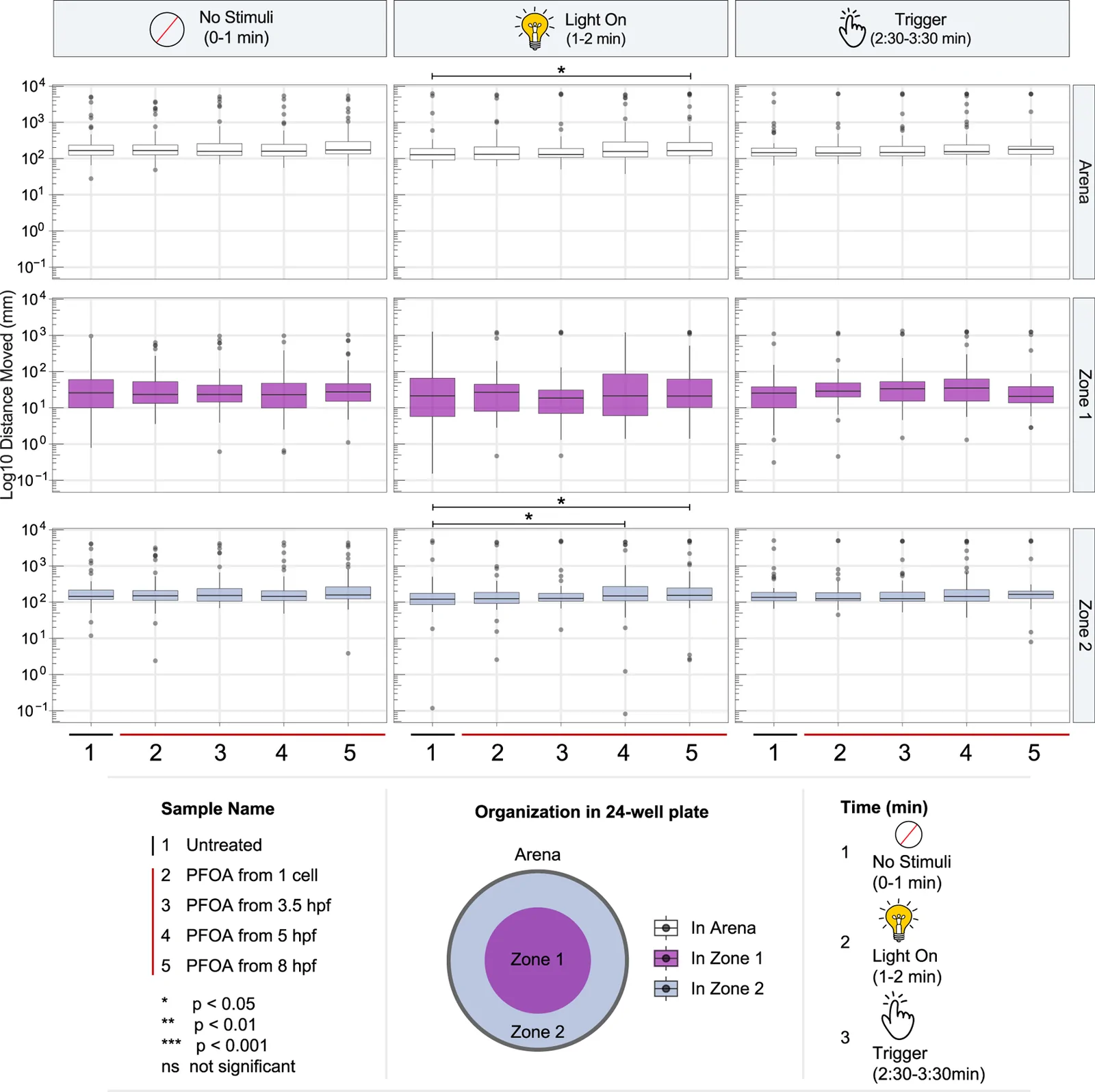
PFOA-treated larvae exhibit anxiety-like behavior in response to stress stimuli. Distance moved by zebrafish larvae (mm) was measured over time (minutes) and analyzed across three regions: Arena (total distance moved), Zone 1 (center), and Zone 2 (periphery). Larvae were individually placed in a 24-well plate (N = 24 per condition). Each well was divided into Zone 1 (center) and Zone 2 (periphery) using EthoVision software, and same boundaries used across all samples. Behavioral activity was recorded across 3 minutes: 0–1 min - baseline without stimulus, 1–2 min - light stimulus, and 2:30–3:30 min - mechanical vibration stimulus. A 30 s no stimuli was added between light and trigger stimulation. Box plots show the total distance moved in each area. Asterisks denote statistical significance when comparing distance moved between zone 2 compared to zone 1: * equals *p* < 0.05, ** equals p < 0.01, *** equals *p* < 0.001, and ns denotes no significant difference in movement.

Heatmap visualizations generated using EthoVision software, in which blue shading depicts regions of movement within the 24-well plate setup, revealed marked increases in both total distance traveled and movement velocity in larvae exposed to PFOA beginning at the 1-cell, 3.5 hpf, 5 hpf, and 8 hpf developmental stages (Figure 6; Supplementary Figures S5–S6). Overall, most PFOA-treated larvae exhibited significantly greater locomotor activity compared to untreated controls, consistent with a hyperactivity-like phenotype, with the strongest and most statistically significant effects observed in the 8 hpf exposure group (Figure 7).

In addition to increased locomotor activity, PFOA-treated larvae displayed a pronounced preference for the periphery of the well, commonly referred to as “wall-hugging” behavior, relative to untreated larvae. In zebrafish behavioral models, this response is frequently interpreted as an anxiety-like phenotype. Zone analysis further supported this observation, showing significantly greater movement within zone 2, the peripheral region of the well, particularly in larvae exposed beginning at 5 hpf and 8 hpf following light stimulation. The enhanced peripheral swimming behavior observed after light exposure suggests that early embryonic PFOA exposure may alter stress-response and sensory-processing pathways, leading to heightened anxiety-like behavior that persists and can be observed at the larval stage.

## Discussion

This study demonstrates that exposure to perfluorooctanoic acid (PFOA), a persistent and bioaccumulative PFAS compound, disrupts zebrafish development with exposure in a stage-dependent manner during the maternal-to-zygotic transition (MZT). Although embryo viability at 24 hpf was not significantly affected, PFOA exposure resulted in multiple developmental and behavioral abnormalities, including trends of reduced tail twitching response, significant transcriptional dysregulation, and anxiety-like locomotor behaviors. These findings indicate that this physiological exposure of PFOA can alter developmental programming without causing overt embryonic lethality, supporting the concept that early embryogenesis represents a sensitive window for environmental toxicant exposure.

A major finding of our study is that the developmental timing of exposure, rather than exposure duration alone, determines the magnitude and nature of the molecular response. The maternal-to-zygotic transition is characterized by extensive maternal RNA degradation, zygotic genome activation (ZGA), chromatin remodeling, enhancer activation, and establishment of developmental gene regulatory networks that coordinate embryonic patterning and organogenesis (Akdogan-Ozdilek et al., 2020). As these regulatory processes occur within discrete developmental windows, environmental perturbations during MZT have the potential to produce stage-specific molecular consequences. Consistent with this concept, embryos exposed to PFOA beginning at 3.5 and 8 hpf exhibited the greatest transcriptomic perturbations, indicating that distinct phases of the MZT represent periods of increased transcriptional susceptibility to environmental exposure.

Rather than selecting genes solely based on differential expression previously exposed with exposure, our targeted qRT-PCR panel was designed to evaluate conserved developmental regulators that govern biological processes occurring during the MZT and early organogenesis. *Wnt3a* and *sfrp5* were selected because Wnt signaling is essential for gastrulation, neural induction, and anterior-posterior axis formation (Patapoutian and Reichardt, 2000). *Hoxb1b*, *vox*, and *cdx4* were chosen because they regulate dorsal-ventral patterning, posterior body specification, and hindbrain segmentation, respectively, and therefore serve as markers of embryonic patterning established during early development (Imai et al., 2001; McClintock et al., 2002; Skromne et al., 2007). *Pa2g4a* and *atm* were included to assess cell proliferation and activation of DNA damage responses (Khanna et al., 2001; Liu et al., 2006), whereas *cited4b* was examined as a marker associated with muscle differentiation and neuromuscular development (Devakanmalai et al., 2013). Collectively, these genes represent developmental pathways known to be highly active during the MZT and early embryogenesis and were selected to determine whether PFOA disrupts critical developmental signaling networks rather than isolated molecular targets.

Our observations are consistent with previous studies demonstrating that developmental PFAS exposure disrupts highly conserved signaling pathways involved in embryonic patterning, neurodevelopment, and cellular homeostasis (Chen et al., 2026; Paddayuman et al., 2025). Altered Wnt signaling, homeobox gene expression, oxidative stress pathways, and DNA damage responses have been reported following PFOA and other PFAS exposures in zebrafish and mammalian model systems, supporting the idea that these developmental pathways represent common molecular targets of PFAS toxicity (Ko et al., 2026; Rericha et al., 2023; Winstanley et al., 2026). However, most previous studies have evaluated continuous exposures or single developmental time points (Chaojie et al., 2026; Natha et al., 2026), making it difficult to determine whether specific stages of embryogenesis exhibit differential susceptibility. By initiating exposure at discrete developmental stages spanning the MZT, our study demonstrates that developmental timing itself is a critical determinant of the transcriptional response to PFOA.

Interestingly, these developmental regulators exhibited pronounced stage-specific responses. Components of the Wnt signaling pathway, including *wnt3a* and *sfrp5*, were significantly downregulated, suggesting disruption of signaling pathways required for early neural development and embryonic patterning. Likewise, *vox* and *cdx4* expression was reduced following specific exposure windows, whereas *hoxb1b* remained relatively unchanged, indicating that not all developmental transcription factors are equally sensitive to PFOA. Genes regulating proliferation and cellular stress also exhibited stage-dependent responses, with reduced *pa2g4a* expression and increased atm expression suggesting impaired proliferative capacity accompanied by activation of DNA damage response pathways. Increased *cited4b* expression may reflect compensatory changes in muscle differentiation or broader dysregulation of neuromuscular developmental programs.

An important observation was that several genes, including *vox*, *pa2g4a*, and *cited4b*, were significantly altered following exposure beginning at 3.5 or 5 hpf, but showed comparatively limited responses when exposure was initiated at the 1-cell stage, despite the longer exposure duration. One explanation could be that cleavage-stage embryos rely predominantly on maternally deposited RNAs and proteins before widespread zygotic transcription begins. Consequently, perturbations occurring before ZGA may initially be buffered by maternal regulatory mechanisms, delaying measurable transcriptional consequences. In contrast, the blastula stages coincide with extensive chromatin remodeling, enhancer activation, maternal transcript clearance, and establishment of zygotic transcriptional programs. Perturbations during this interval are therefore more likely to directly influence developmental gene regulatory networks, resulting in the pronounced transcriptional changes observed at 3.5 and 5 hpf. By 8 hpf, many early patterning events have already been established; however, PFOA exposure may still interfere with ongoing transcriptional activation and maintenance of zygotic gene expression. This may contribute to the greater degree of transcriptional downregulation observed following exposure initiated at this stage. Although these mechanisms require direct experimental validation, our findings support the hypothesis that developmental susceptibility is governed by the regulatory state of the embryo rather than exposure duration alone.

Global transcriptomic profiling further supports this interpretation. The stage-specific transcriptomic responses closely mirror the biological events occurring throughout the MZT. At the 1-cell stage, enrichment of pathways related to lipid storage, RNA processing, and oxidative metabolism likely reflects disruption of maternal regulatory and metabolic programs that support development before ZGA. During the blastula stages (3.5–5 hpf), enrichment of pathways involved in RNA processing, DNA damage responses, spliceosome function, cell proliferation, and p53 signaling suggests interference with the extensive transcriptional reorganization accompanying zygotic genome activation. These responses may represent compensatory mechanisms that preserve genome integrity while developmental regulatory networks are being established. By 8 hpf, after completion of the MZT and during early organogenesis, the transcriptomic response shifted toward suppression of oxidative phosphorylation, ATP metabolism, mitochondrial function, and activation of immune-related pathways, suggesting that PFOA-induced stress progresses from disruption of developmental regulatory programs to broader impairment of cellular metabolism and tissue differentiation. One potential explanation is that embryos at each developmental stage possess distinct chromatin accessibility landscapes and enhancer repertoires. Environmental perturbation during periods of extensive chromatin remodeling may therefore alter upstream cis-regulatory elements or transcription factor activity, ultimately producing the stage-specific downstream transcriptional responses observed in this study. Although enhancer activity was not directly examined, our findings are consistent with disruption of developmental gene regulatory networks with exposure during MZT.

Similar stage-dependent responses have been described for other developmental toxicants, where exposure during periods of extensive chromatin remodeling and zygotic genome activation produces disproportionately large transcriptional consequences compared with exposures outside these windows (Vliet et al., 2019; Wu et al., 2025; Yu et al., 2023). Likewise, previous transcriptomic studies of PFOA and other PFAS have reported alterations in oxidative metabolism, mitochondrial function, immune signaling, and neuronal development (Gaillard et al., 2025; Goyal et al., 2026; Kebieche et al., 2025; Ko et al., 2026; Luebker et al., 2005; Olsen et al., 2007; Qi et al., 2023; Rawtani et al., 2026; Satbhai et al., 2025; Tursi et al., 2024). Our data extend these observations by demonstrating that many of these pathways are differentially affected depending on the developmental stage at which exposure occurs, suggesting that the timing of exposure determines which biological processes are most susceptible to PFAS-induced disruption.

The behavioral abnormalities observed at 5 dpf demonstrate that these early molecular perturbations persist beyond embryogenesis and are accompanied by functional consequences. Behavioral assays are inherently sensitive to environmental variables, and although all efforts were made to maintain consistent experimental conditions and imaging procedures across treatment groups, we cannot completely exclude the possibility that minor uncontrolled factors (e.g., subtle fluctuations in room temperature or humidity) contributed to some variability in the behavioral responses. Nevertheless, we did observe that PFOA-exposed larvae displayed increased locomotor activity, heightened responsiveness to light stimuli, and pronounced thigmotaxis, behaviors commonly associated with altered neural circuit development and anxiety-like phenotypes in zebrafish. Together with the observed changes in developmental signaling pathways and genes associated with neuromuscular differentiation, these findings suggest that disruption of developmental programming during the MZT can influence nervous system function later in development. Together with the observed changes in developmental signaling pathways and genes associated with neuromuscular differentiation, these findings suggest that disruption of developmental programming during the maternal-to-zygotic transition can have persistent effects on nervous system function and behavior later in development.

Collectively, our findings are consistent with an expanding body of literature demonstrating that developmental PFAS exposure perturbs neurodevelopment, oxidative metabolism, and transcriptional regulation (Kebieche et al., 2025; Lagostena et al., 2025). While previous studies have identified developmental toxicity following continuous or single-window PFAS exposures, our study demonstrates that the developmental timing of exposure is itself a critical determinant of the molecular response. Rather than eliciting a uniform toxicological effect, PFOA induces distinct transcriptional programs depending on whether exposure occurs before, during, or after the maternal-to-zygotic transition. These findings identify the MZT as a critical developmental window during which disruption of developmental gene regulatory networks may alter embryonic patterning, tissue differentiation, and subsequent neurobehavioral outcomes. The stage-specific transcriptional responses observed here further support the hypothesis that PFOA may interfere with coordinated developmental regulatory programs, potentially through disruption of upstream cis-regulatory elements and enhancer-mediated transcription, although this will require direct experimental validation. Future studies integrating transcriptomic, epigenomic, and functional approaches will be important for defining these mechanisms and identifying the developmental regulatory networks most vulnerable to PFAS exposure. Collectively, our findings provide mechanistic insight into how PFAS exposure during early embryogenesis can alter neurodevelopmental trajectories, consistent with epidemiological studies (Saha et al., 2025; Yao et al., 2023) linking prenatal PFAS exposure to neurobehavioral and metabolic disorders in humans.

## Data Availability

All raw and processed data files along with metadata are available online at the GEO database. The accession number is GSE343095. https://www.ncbi.nlm.nih.gov/geo/query/acc.cgi?acc=GSE343095.

## Supplementary material

SUPPLEMENTARY FIGURE S1
**(A)** Bar graph of Gene Ontology (GO) term enrichment analysis of differentially expressed genes (DEGs) identified in 1-cell exposure treatment compared to untreated control. Enrichment is shown across three categories: Biological Process (BP), Cellular Component (CC), and Molecular Function (MF). **(B)** Dot plot of KEGG pathway enrichment analysis identified in 1-cell exposure treatment compared to untreated control. Dot color represents statistical significance, while dot size indicates the number of DEGs mapped to each KEGG pathway.

SUPPLEMENTARY FIGURE S2
**(A)** Bar graph of Gene Ontology (GO) term enrichment analysis of differentially expressed genes (DEGs) identified in 3.5 hpf exposure treatment compared to untreated control. Enrichment is shown across three categories: Biological Process (BP), Cellular Component (CC), and Molecular Function (MF). **(B)** Dot plot of KEGG pathway enrichment analysis identified in 3.5 hpf exposure treatment compared to untreated control. Dot color represents statistical significance, while dot size indicates the number of DEGs mapped to each KEGG pathway.

SUPPLEMENTARY FIGURE S3
**(A)** Bar graph of Gene Ontology (GO) term enrichment analysis of differentially expressed genes (DEGs) identified in 5 hpf exposure treatment compared to untreated control. Enrichment is shown across three categories: Biological Process (BP), Cellular Component (CC), and Molecular Function (MF). **(B)** Dot plot of KEGG pathway enrichment analysis identified in 5 hpf exposure treatment compared to untreated control. Dot color represents statistical significance, while dot size indicates the number of DEGs mapped to each KEGG pathway.

SUPPLEMENTARY FIGURE S4
**(A)** Bar graph of Gene Ontology (GO) term enrichment analysis of differentially expressed genes (DEGs) identified in 8 hpf exposure treatment compared to untreated control. Enrichment is shown across three categories: Biological Process (BP), Cellular Component (CC), and Molecular Function (MF). **(B)** Dot plot of KEGG pathway enrichment analysis identified in 8 hpf exposure treatment compared to untreated control. Dot color represents statistical significance, while dot size indicates the number of DEGs mapped to each KEGG pathway.

SUPPLEMENTARY FIGURE S5 Line graphs depicting the total distance moved by untreated and PFOA-exposed larvae over 3-min and 30-s time bins. Locomotor activity was measured as total distance traveled in mm within the arena of each well. The arena was divided into two regions: zone 1 (center) and zone 2 (periphery) to assess spatial movement patterns and anxiety-like behavior.

SUPPLEMENTARY FIGURE S6 Line graphs depicting the movement of larvae or their velocity for untreated and PFOA-exposed larvae over 3-min and 30-s time bins. Locomotor activity was measured as total distance traveled in mm over time in seconds (mm/s) within the arena of each well. The arena was divided into two regions: zone 1 (center) and zone 2 (periphery) to assess spatial movement patterns and anxiety-like behavior.
