# Supplementary material for: The maternal-to-zygotic transition is a critical window for PFOA-induced disruption of developmental programming
Source: Front Cell Dev Biol. 2026 Aug 26;14:1887853. doi: 10.3389/fcell.2026.1887853 (PMC13553801; doi:10.3389/fcell.2026.1887853)

Supp Figure 3

A) GO term analysis for PFOA exposure from 5 hpf compared against untreated separated by categories

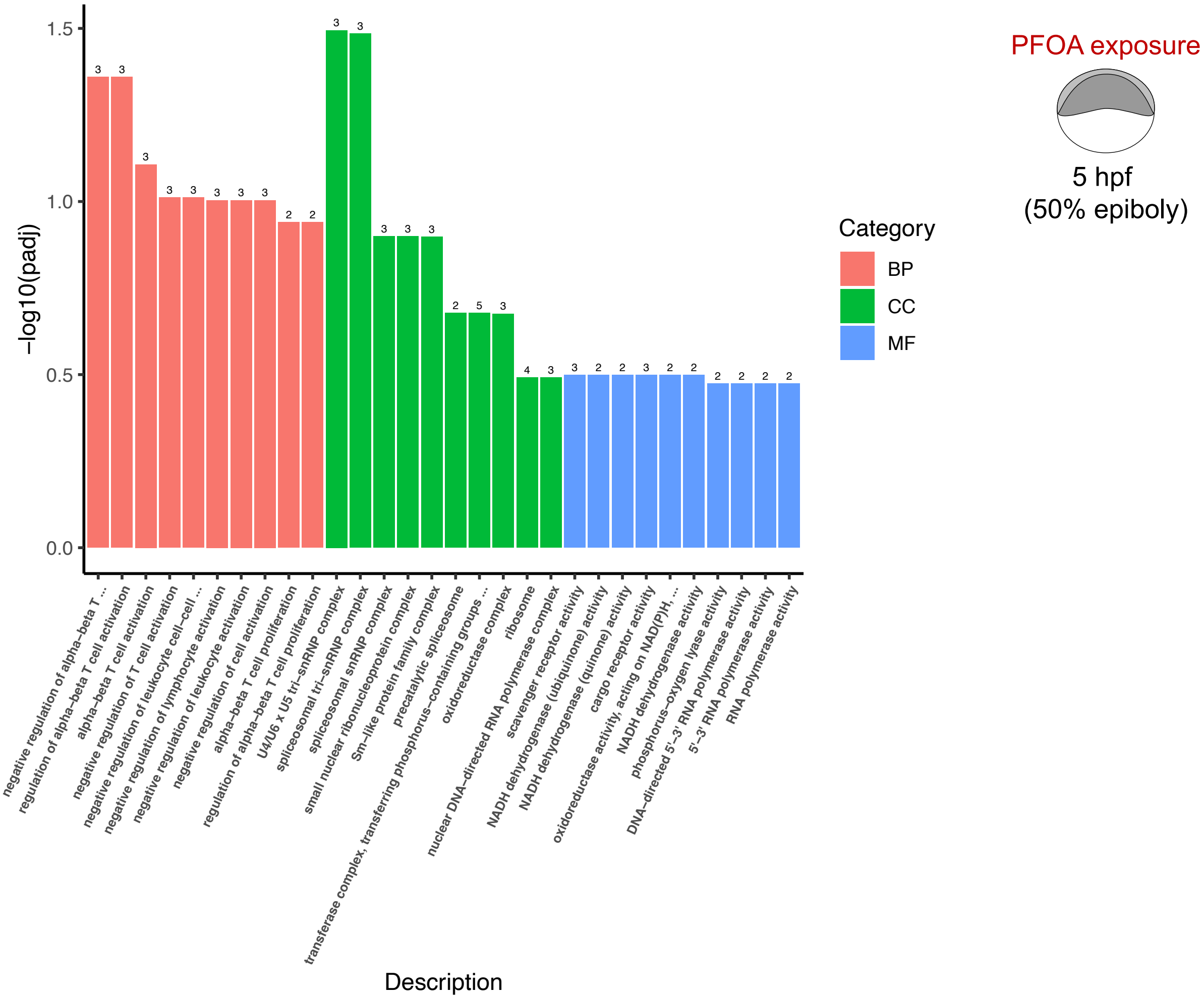

B) Kegg pathway analysis for PFOA exposure from 5 hpf compared against untreated

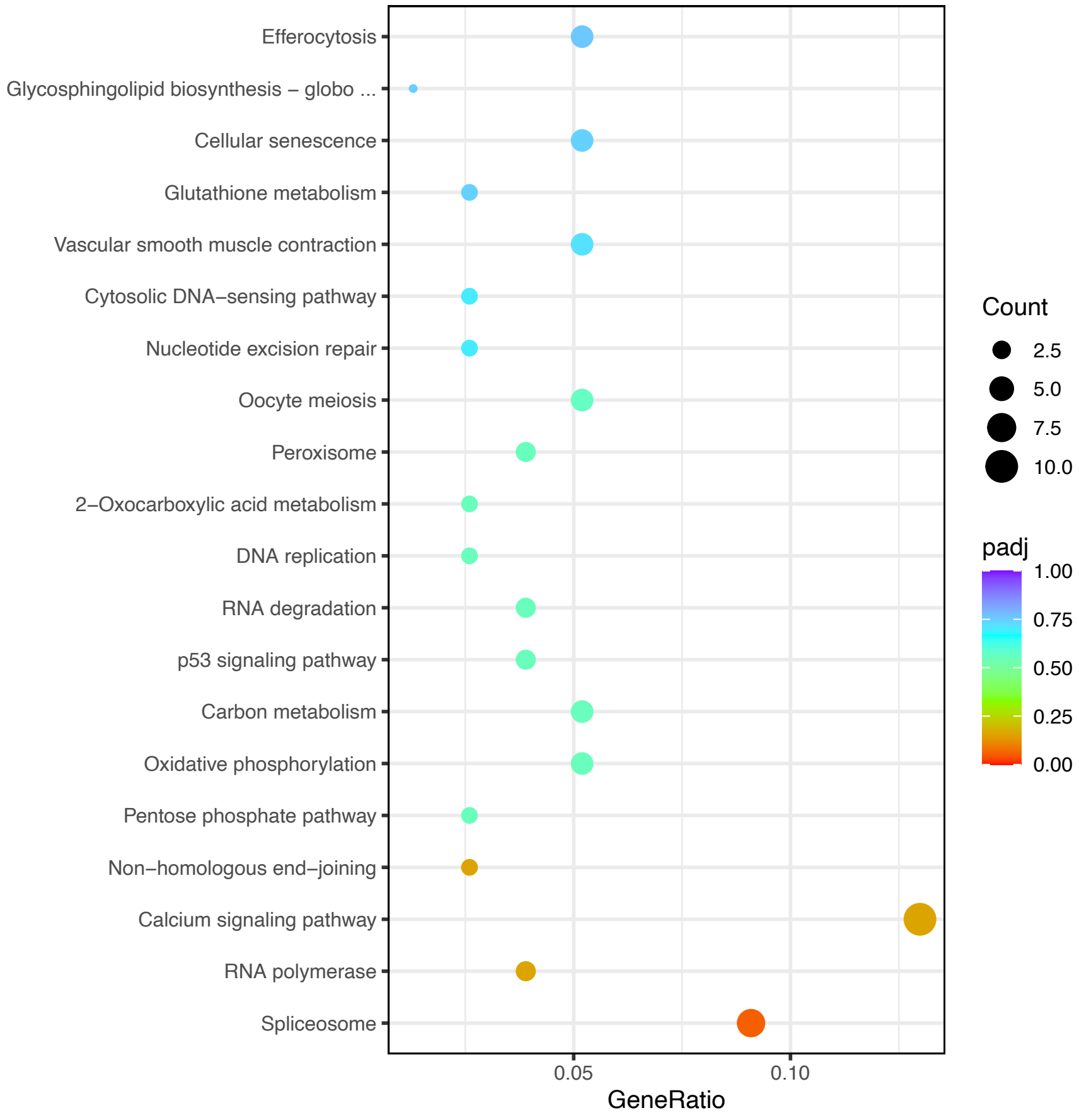

Supplement: Supplementary file 3 [file DataSheet3.pdf]
