# Supplementary material for: The maternal-to-zygotic transition is a critical window for PFOA-induced disruption of developmental programming
Source: Front Cell Dev Biol. 2026 Aug 26;14:1887853. doi: 10.3389/fcell.2026.1887853 (PMC13553801; doi:10.3389/fcell.2026.1887853)
Supplement: Supplementary file 6 [file DataSheet6.pdf]

Supp Figure 6

Line graphs for Velocity differences in Arena, Zone 1, and Zone 2 for different PFOA exposures

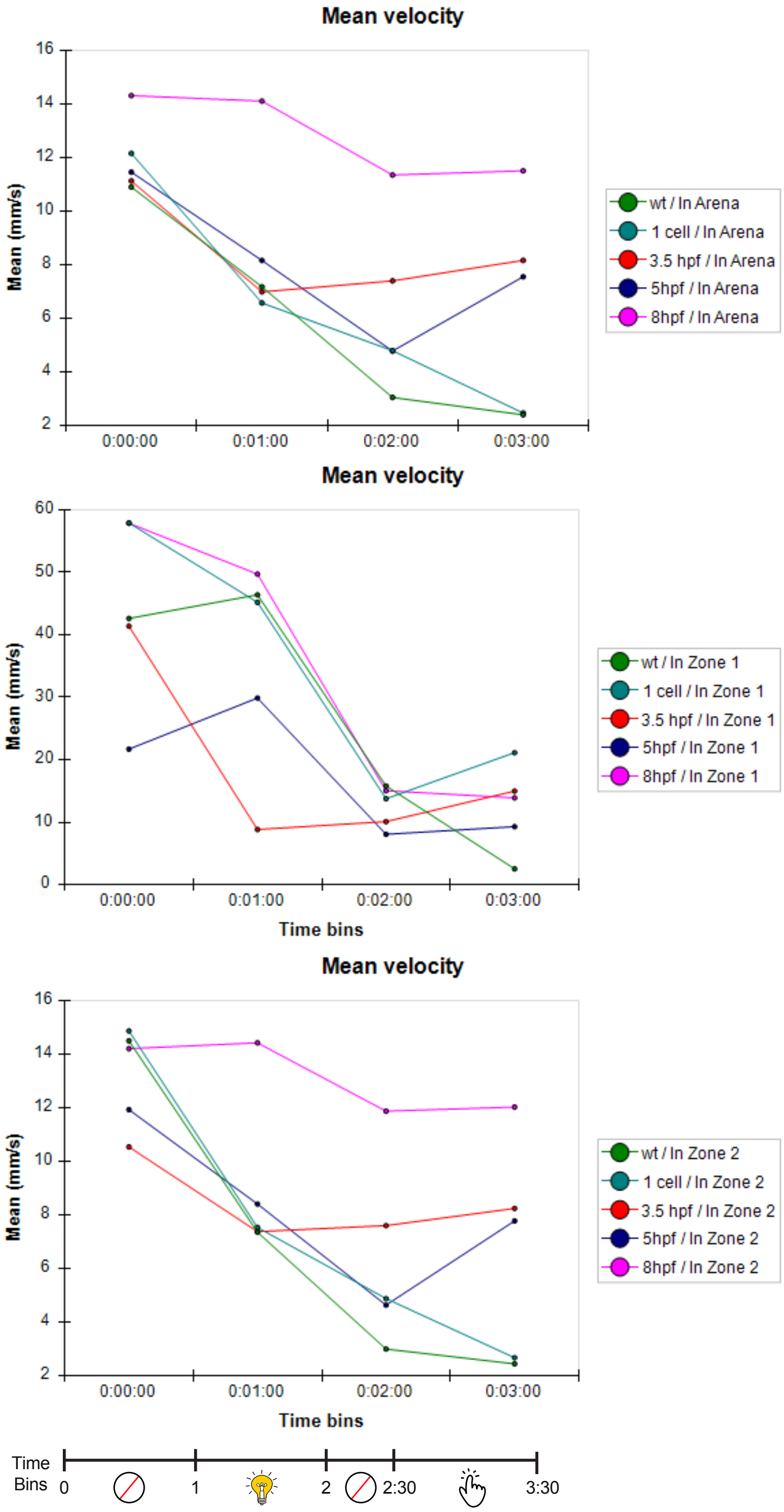

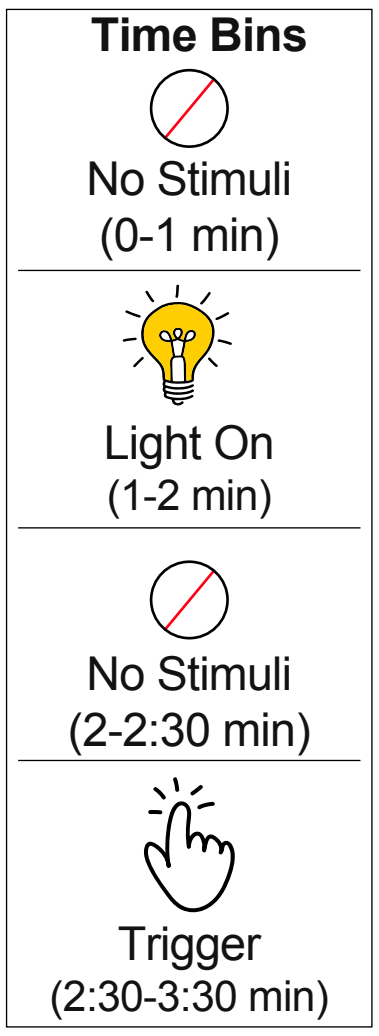

Organization in 24-well plate

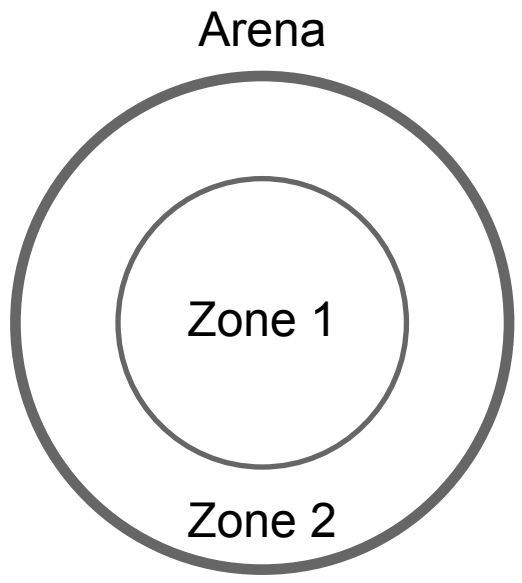

Sample Name

WT - Untreated

1 Cell - PFOA treatment from 1 cell

3.5 hpf - PFOA treatment from 3.5 hpf

5 hpf - PFOA treatment from 5 hpf

8 hpf - PFOA treatment from 8 hpf
